# Mechanical Properties, Critical Length, and Interfacial Strength of Seven-Islands-Sedge Fibers (*Cyperus malaccensis*) for Possible Epoxy Matrix Reinforcement

**DOI:** 10.3390/polym14183807

**Published:** 2022-09-12

**Authors:** Lucas de Mendonça Neuba, Raí Felipe Pereira Junio, Andressa Teixeira Souza, Matheus Pereira Ribeiro, Pedro Henrique Poubel Mendonça da Silveira, Thuane Teixeira da Silva, Artur Camposo Pereira, Sergio Neves Monteiro

**Affiliations:** Department of Materials Science, Military Institute of Engineering-IME, Praça General Tíburcio, 80, Urca, Rio de Janeiro 222290-270, Brazil

**Keywords:** seven-islands-sedge fiber, cyperus malaccensis, ultimate tensile test, XRD, pullout test

## Abstract

The growing concern about the limitation of non-renewable resources has brought a focus on the development of environmentally sustainable and biodegradable composite materials. In this context, a trend in the development of natural fibers used as a reinforcement in composites is ever-increasing. In this work, for the first-time, fibers extracted from the seven-islands-sedge plant (*Cyperus malaccensis*) have been characterized by X-ray diffraction (XRD) to calculate the crystallinity index and the microfibrillar angle (MFA). Also, an evaluation of the ultimate tensile strength by diameter intervals has been investigated and statistically analyzed by both the Weibull method and the analysis of variance (ANOVA). Moreover, the maximum deformation and tensile modulus have been found from the data acquired. Pullout tests have been conducted to investigate the critical length and interfacial strength when sedge fibers, are incorporated into epoxy resin matrix. Microstructure analysis by scanning electron microscopy (SEM) was performed to observe the mechanism responsible for causing rupture of the fiber as well as the effective fiber interfacial adhesion to the epoxy matrix.

## 1. Introduction

In recent years, a growing concern for the limitation of non-renewable resources has directed the world to focus on the development of environmentally sustainable and biodegradable composite materials. Environmental legislation and industrial demands worldwide have contributed to the emergence of new studies in search of natural materials that replace synthetic ones, thus reducing the dependence on non-renewable raw materials [1,2].

Currently, the interest in the use of polymer matrix composites reinforced with natural lignocellulosic fibers (NLFs) has increased. These composites do not require much energy for processing and have a relatively low cost. In addition, the processing of NLFs requires less energy compared to synthetic fibers such as glass and carbon [3]. Their importance has been recognized since ancient times, when these fibers were already used in various applications, due to their specific properties and applications [4,5]. Currently, NLFs have successfully replaced synthetic fibers, which are more expensive and not sustainable. Indeed, NLFs have many advantages because they are renewable, biodegradable, low cost, and display good mechanical properties [6,7]. Due to demand and sustainability issues, the interest for the development of environmentally friendly materials such as NLFs composites has been increasing among researchers [8,9], and they have also been industrially applied [10,11,12].

NLFs might be compared to a natural composite material consisting basically of three distinct phases: one crystalline phase and two amorphous phases. The crystalline phase is microfibrillar cellulose, considered as the reinforcing phase, while lignin and hemicellulose are considered as the amorphous matrix phases [13,14,15,16,17]. Cellulose provides the largest portion by weight of natural fibers, so the percentage of cellulose in the fiber directly affects properties such as density, Young’s modulus, and tensile strength of the fibers. Each cellulose repeating unit contains three hydroxyl groups; these groups, along with their ability to form hydrogen bonds, act as the primary means of determining the physical properties of cellulose [18].

Regarding the study of NLFs properties, one fiber with potential for composite reinforcement is the seven-islands-sedge fiber, extracted from the plant *Cyperus malaccensis Lam.* This plant, belonging to the family *Cyperaceae*, is used as a raw material for making tatami mats, with its cultivation being predominant in China and Japan [19,20]. The *Cyperus malaccensis* plant has a sharp, triangular, and long stem, an absence of nodes, and about 2–3 leaves at the tip of the stem. Harvesting occurs when the plant reaches a height of about 1.5 m after its stem has been cut several times to ensure uniform stem thickness [21].

Seven-islands-sedge fibers, sedge fibers for short, have potential application as reinforcement in polymer matrix composites. However, little information is available in the literature regarding this fiber in composites. Neuba et al. [22] employed sedge fibers as reinforcement in epoxy matrix composites incorporated with 10, 20, and 30 vol% to study their mechanical, thermal, and ballistic properties. The addition of sedge fibers promoted an increase in the impact strength of composites compared to plain epoxy, but for the tensile strength, the result was not satisfactory.

Based on the lack of information about this fiber, the present work aimed to provide a deeper evaluation of the tensile strength of sedge fibers by means of diameter dependence. Moreover, the crystallinity and microfibrillar angle by X-ray diffraction (XRD), as well as the critical length and interfacial strength of the sedge fibers by pullout in epoxy matrix, were also investigated.

## 2. Materials and Methods

### 2.1. Materials

**Figure 1:**
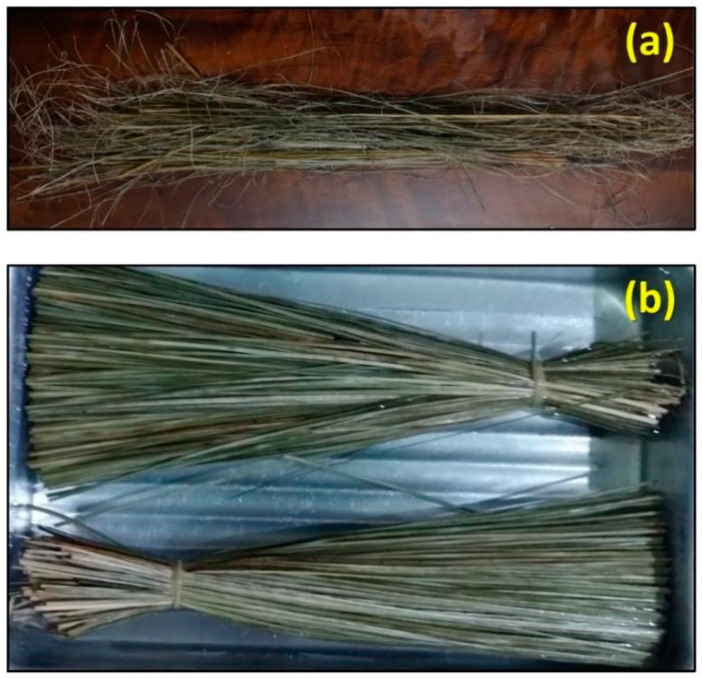
(**a**) Sedge fibers as-received; (**b**) sedge fibers after cutting, washing, drying, and measuring, ready for characterization.

The sedge fibers investigated in this paper were extracted from a mat supplied by the company Artevale, Brazil. The as-received fiber bundle used in the study is shown in Figure 1a. The fibers were manually cut to a length of 150 mm. After cutting, the fibers were washed with DI water and immersed for 24 h. Then, they were dried in an oven at 70 °C for 24 h, as shown in Figure 1b, ready for characterization.

The manual extraction of sedge fibers was performed using a sharp stiletto to carefully separate them from the as-received stalks, which were originally separated by the commercial supplier from the plant stem. This process is similar to that used to extract fibers from the rigid culm of bamboo [23]. In the first step, the rigid as-received stalk of the *Cyperus malaccensis* plant is immersed in water to soften and facilitate the extraction of individual sedge fibers. Sedge fibers extracted in this way, like bamboo [23], ubim [24], buriti [16], and caranan [25], are prone to be thicker, with cross-section dimensions within tenth of mm, while plants like sisal ramie and jute, from which fibers can be easier separated, have dimensions within hundredth of mm dimensions [16]. Despite different diameters (average cross-section dimension), all carefully extracted sedge fibers are single ones, with well-known internal structure of walls with spiraling crystalline cellulose microfibrils embedded in amorphous lignin and hemicellulose matrix. Detailed cross-sectional observation confirmed each extracted sedge fiber with only one lumen, which is evidence of individuality. Initially, the length of each sedge fiber corresponds to that of the stalk, ~900 mm, cut by the supplier. For composite preparation, this initial sedge fiber length was reduced to 150 mm each.

### 2.2. X-ray Diffraction (XRD)

The crystallinity index (*Ic*) and the microfibrillar angle (MFA) of the sedge fibers were calculated from the diffractogram obtained by X-ray diffraction (XRD). To calculate the fiber *Ic*, the proposed method by Segal et al. [26] was used, in which the crystallinity is given by the difference of the peak relative to the (0 0 2) plane, by the peak of the amorphous halo in the diffractogram:

(1) $$I_{C\;}=\frac{I_{0\;0\;2}-I_{am}}{I_{0\;0\;2}}\times 100\;\%$$

A PANalytical X’pert Pro MRD diffractometer, with cobalt anode (0.1789 nm), was used with the following parameters to obtain the diffractogram: scan rate of 0.05 (2θ/s), scan from 5° to 75°, 40 mA, and 40 kV. For the calculation of the MFA, the method used was based on the methodology proposed by Cave [27] and replicated in other papers [28,29,30]. The MFA value was determined from the Gauss curve of the second peak ((0 0 2) plane), along with the first- and second-order derivatives of the Gauss curve. This determination used Origin Pro Software, adopting the following steps: (i) obtaining the diffractogram and removing the baseline from the diffractogram; (ii) determining the Gaussian associated with the second peak; (iii) plotting the first and second Gaussian derivatives; and (iv) merging the three curves into a single graph. The value of *T*, which is the angle between the line drawn from the center of the peak of the Gaussian curve to the point of intersection between the first and second derivatives, was used in the calculation responsible for determining the *MFA*, calculated by:

(2) $$MFA=-12.198\;T^{3}+113.67\;T^{2}-348.4\;T+358.09$$

### 2.3. Tensile Tests

**Figure 2:**
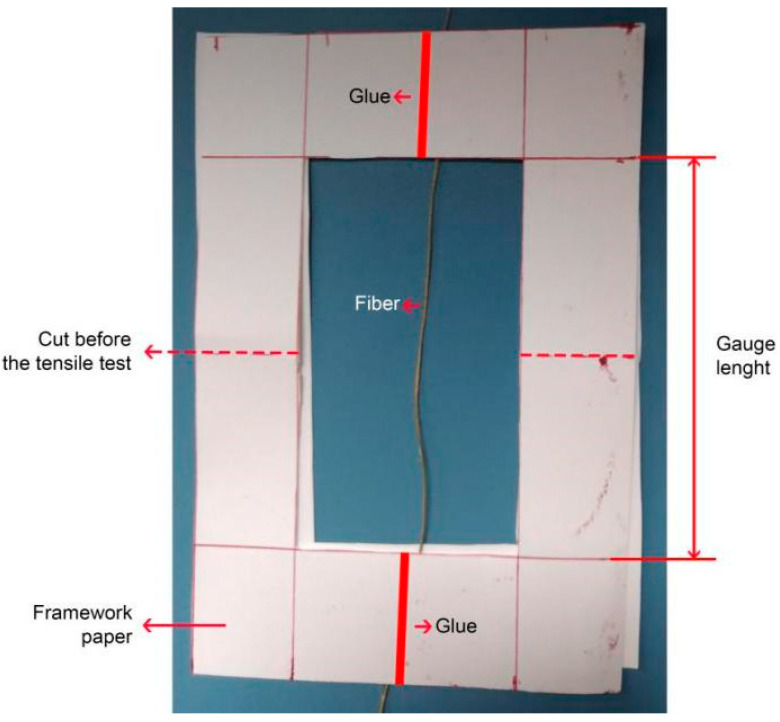
Apparatus used for the tensile test of sedge fibers.

Tensile tests of sedge fibers were performed at room temperature to analyze the variation of the tensile strength in 6 different diameter intervals. As such, it was possible to obtain an average tensile strength ratio directly related to the cross-section diameter of each interval. The preparation of the samples for the fiber tensile test was performed according to ASTM D2101-95 [31]. The samples were tested with the ends of the fibers protected by adhesive tape, preventing the fibers from slipping between the gripper of the equipment and crushing them. Paper frames, as shown in Figure 2, assisted in the protection of the fibers during testing. This guaranteed good adhesion when in contact with the equipment’s gripper, avoiding fiber slippage during testing and premature fiber breakage. The tests were conducted in a universal machine EMIC. The strain rate employed was 0.9 mm/min, with a gauge length of 90 mm.

The tensile strength values obtained were statistically interpreted by the Weibull Analysis software, which provided the corresponding scale parameter (*θ*), shape parameter (*β*), and precision fit parameters (R²). The software is based on the cumulative Weibull frequency distribution function (*F*(*x*)), given by Equation (3), in which *β* is a linear slope.

(3) $$ln\;ln\;\left[ln\;ln\;\left(\frac{1}{1-F\left(x\right)}\right)\;\right]=\beta \;ln\;ln\;\left(x\right)-\left[\beta \;ln\;ln\;\left(\theta \right)\right]$$

Analysis of variance (ANOVA) was performed, using the F-test in order to verify significant differences between the means of the results obtained, in addition to the confidence levels used in all tests being 95%. The Tukey test was also used for each sample group; this test is known as an honestly significant difference (HSD). The aim is to quantitatively evaluate each of the fiber percentages used, where the hypothesis of equality is rejected based on the HSD, given by Equation (4). The term *q* is the total range studied, which is a function of the degree of freedom (DF) of the residual and the number of treatments; *MSE* is the mean square error, and *r* is the number of replicates of each treatment [32].

(4) $$\mathrm{HSD}=q\cdot \sqrt{\frac{MSE}{r}}$$

### 2.4. Pullout Tests

In the fiber pullout test, a total of 80 samples was used. For each cylindrical sample, a single fiber was inserted into the epoxy resin matrix. The groups were divided by considering different embedding lengths. For each group, ten samples were separated; considering L as the length where L = 2, 4, 6, 8, 10, 15, 20, and 25 mm. The mold adopted for the matrix was cylindrical-shaped, 50 mm long, and 8 mm in diameter. The pull-out test was performed at a strain rate of 0.75 mm/min using a load cell of 25 kN in an Instron model 3365 universal machine. Following the model proposed by Kelly and Tyson [33], it was possible to obtain the critical length and relate it to the sedge fiber/epoxy interfacial strength.

### 2.5. Scanning Electron Microscopy (SEM)

The sedge fiber surface and epoxy embedded fibers were analyzed using a Quanta FEG 250, Fei microscope (Hillsboro, DC, USA). The equipment operated with a secondary electron detector at an acceleration voltage between 9 and 10 kV. The fibers were gold-coated in the Leica Ace 600 equipment (Wetzlar, Germany) in order to create an electron current so that images illustrating the mechanisms of failure could be observed with a higher resolution and few artifacts.

## 3. Results and Discussion

### 3.1. XRD Results

**Figure 3:**
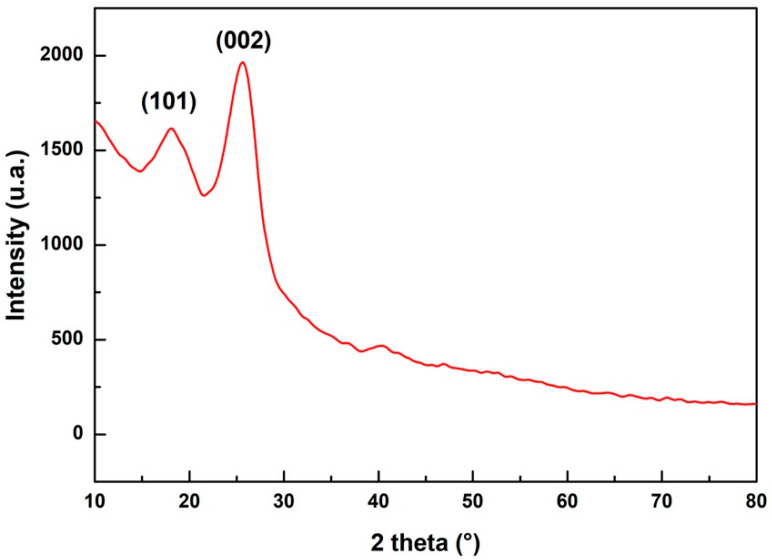
XRD diffractogram of sedge fiber.

Figure 3 shows the diffractogram of the sedge fiber. Note the formation of two reflections: one amorphous halo and one crystalline peak referring to the planes (1 0 1) and (0 0 2) at 18.10 and 25.63°, respectively. The halo (1 1 0) is associated with the incidence of noncellulosic resources like lignin and hemicellulose, whereas the peak in the (0 0 2) plane represents the cellulose content in the natural fiber [34,35]. It has been reported that the peak of the (0 0 2) plane increases and becomes larger in fibers with higher cellulose content [36,37,38].

Using Equation (3), it was possible to calculate the crystallinity of the sedge fiber. A value of *Ic* = 62.47% was found. This crystallinity value of sedge fiber is close to that of other fibers such as vetiver (67%) [39] and areca catechu fiber (66H%) [40]. The crystallinity of sedge fiber proved to be higher than fibers such as sisal [41] and windmill palm fiber [42]. Mayandi et al. [43] observed in *Cyperus pangorei* fiber, which is from the same botanical genus as the seven-islands-sedge fiber, a halo corresponding to the (1 1 0) plane at 15.7° and to the (2 0 0) plane at 22.1°. The crystallinity index was 41%, showing that fibers of the same genus exhibit a lower crystallinity index.

**Figure 4:**
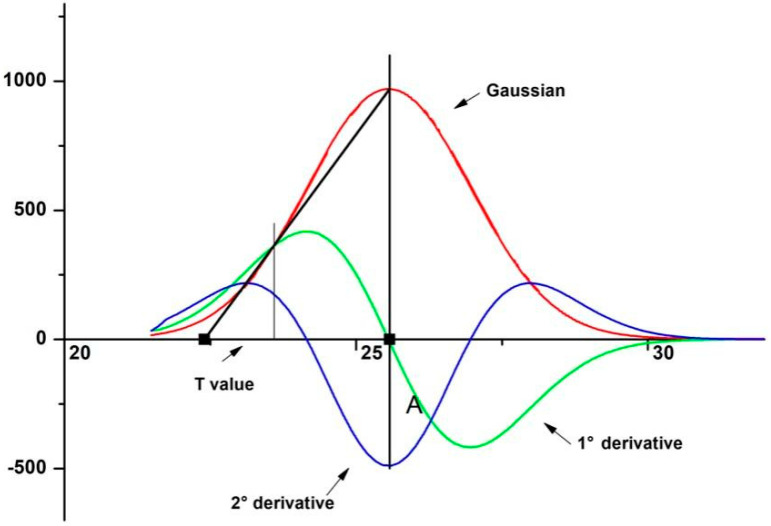
Peak deconvolution used in the microfibrillar angle calculation.

The MFA was obtained from the diffractogram present in Figure 4. As previously stated, the peak in the plane (0 0 2) represents the crystalline fraction of the NLF. From the deconvolution of this peak, the MFA value was obtained, where the lower the MFA value, the better its mechanical properties. The MFA for the sedge found was 7.36°. The lower MFA value, as well as the higher *Ic*, indicate that the tensile strength properties and the modulus of elasticity of the sedge fibers might present superior values when compared to fibers previously studied in the literature [44]. This correction cannot be done exactly, due to several factors that influence the properties of natural fibers, such as moisture content, plant age, fiber diameter, and processing techniques, among others [10].

### 3.2. Tensile Tests Results

Table 1 presents the ultimate tensile strength data obtained from the tensile test related to the diameter intervals, in addition to the Weibull parameters of *β*, R², and the ultimate characteristic tensile stress (*θ*).

Based on the values of the R² parameter present in Table 1, the data present statistical distribution with high accuracy. There is a trend of increasing tensile strength as the fiber diameter is reduced, resulting in a significant difference in the mechanical properties of fibers with larger diameters. Higher tensile strength values in fibers with smaller diameters are found because there is a lower density of defects in these fibers, resulting in better mechanical properties [16]. The characteristic stress of the sedge fibers was found using Weibull statistics. The results indicate a sudden drop in the characteristic ultimate tensile strength between the first and second interval and a tendency of reduction of the stress values up to the largest average diameter interval. The fourth interval showed higher values compared to the last one.

**Figure 5:**
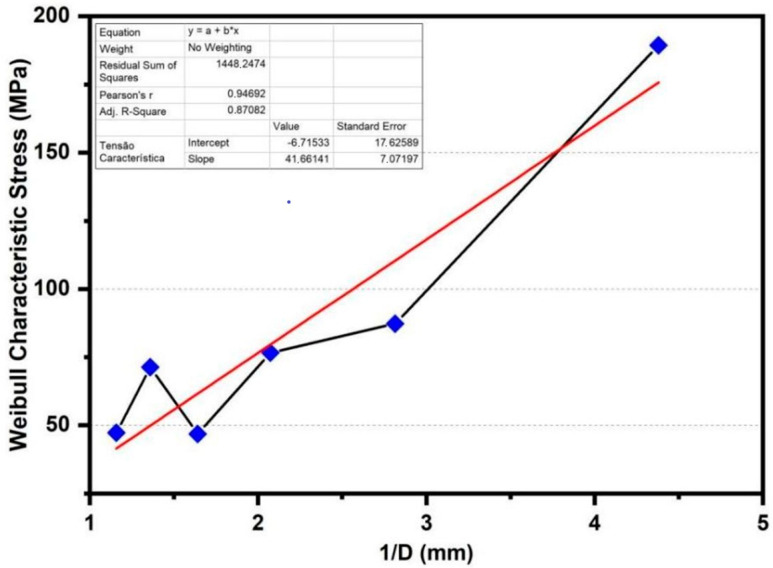
Linear adjustment of ultimate characteristic tensile test for each diameter interval.

A linear approximation was performed using a plot representing the relationship between the characteristic stress and the inverse of the average diameter, shown in Figure 5. 

The mathematical correlation can be seen in Equation (5).

(5) $$\sigma =\frac{41.66141}{D}-6.71533$$

The ultimate tensile strength decreases as fiber diameter increases. This is associated with the higher number of fibrils present in thicker fibers, acting as a failure mechanism that causes premature fiber breakage when compared to thinner fibers, because thinner fibers have fewer defects and are more uniform. The reduction in mechanical properties is still associated with an accumulation of defects within the fiber diameter variations [43]. 

In order to compare the investigated sedge fiber, the Table 2 presents the tensile strength of different NLFs and synthetic fibers. The mean value obtained for the sedge fiber was approximately 75 MPa, which is higher than *Saccharum bengalense* [44]. Moreover, the sedge fiber tensile strength presents similar results to coir (106–270 MPa), which is a globally investigated fiber [45]. While the synthetic fibers depict values more than 10 times higher when compared to sedge [46,47,48,49], the specific values (divided by the density) might be favorable for NLFs. 

### 3.3. Microstructural Analysis

**Figure 6:**
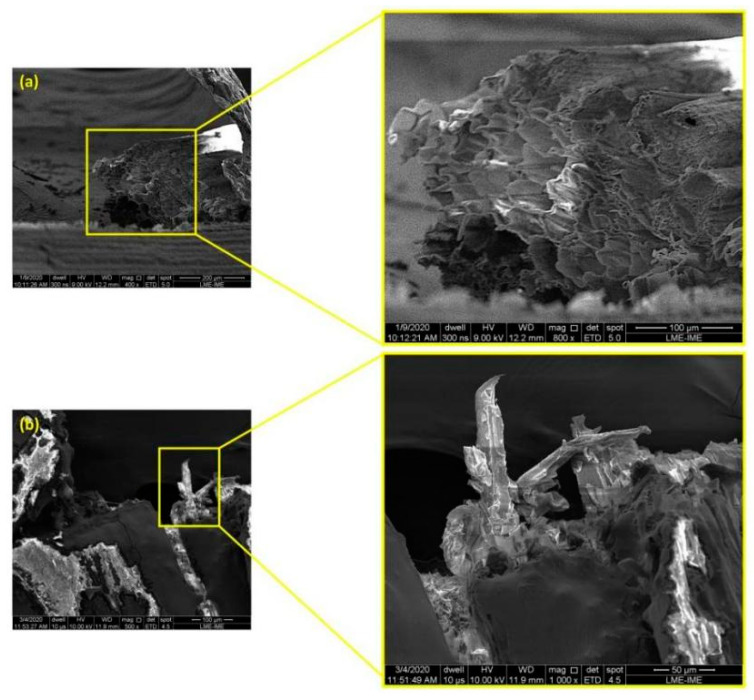
Micrograph of the fracture surface of sedge fiber: (**a**) fracture surface indicating the microfibrils. Magnification 400 and 800×; (**b**) fiber rupture under tensile stress. Magnification 500 and 1000×.

SEM images of sedge fibers are presented in Figure 6. These images corroborate the mechanisms associated with premature failure in fibers with larger diameters, according to the reports presented in the literature [50]. Figure 6a indicates the presence of fibrils inside the fiber and reveals that the sedge fibrils have several voids in their structure, which are found in other NLFs, as reported by Mahdi et al. [51]. These voids act as a premature failure mechanism, directly associated with a low tensile stress property, as analyzed in Table 1. Figure 6b shows the surface of a thicker fiber after performing the tensile test. In this figure, the presence of fibrils can be observed in the fiber cross-section. Indeed, sedge fiber has several regions with voids between fibrils, increasing the probability of the fibers breaking prematurely.

### 3.4. Fiber Diameter and Mechanical Properties Correlation

In order to evaluate the influence of the mean diameter associated with the mechanical properties, an ANOVA analysis was carried out. The results are presented in Table 3.

Based on the significance values in Table 3 after the ANOVA test, the hypothesis that the means are equal at a 5% level is rejected since the statistic indicated that the calculated F value (7.86) is greater than the critical (tabulated) F value (2.62). The *p*-value has a lower value than the 5% significance level, indicating that the results are different for each diameter range. Tukey test was performed with a 95% confidence level to check which range has the best results in terms of tensile strength properties. The results are presented in Table 4.

The results referring to the first average diameter interval show better performance and higher significance (119.65) associated with its final tensile strength value, which is the only one significantly different from the other intervals, due to the fact that the difference found is greater than HSD (72.72). Based on the tensile test results, it is possible to calculate the modulus of elasticity and the maximum percentage elongation of the sedge fibers for each average diameter interval. The average values for each interval are shown in Table 5. An increase in fiber modulus of elasticity is observed as the fiber diameter tends to decrease; this may be directly related to the tensile strength of the fibers having an increase when the average diameter decreases. As for the maximum percentage of elongation, the diameter groups showed close values, except for the group in which the fibers vary from 0.61 to 0.74 mm, showing a reduction of tensile strain.

Following ASTM D 3379-02 [52], the work by Mayandi et al. [43], using fiber of the same botanical genus known as (*Cyperus pangorei*), obtained final tensile strength of 196 ± 56 MPa, percentage elongation of 1.69%, and tensile modulus of 11.6 ± 2.6 GPa of fibers with a diameter ranging between 0.133 ± 0.017 mm. Considering the value obtained from 163.48 MPa for the smallest diameter range from 0.23 to 0.36 mm, it can be concluded that the sedge fibers have mechanical properties with similar values among species of the same botanical genus.

### 3.5. Pullout Results (Interfacial Strength)

**Figure 7:**
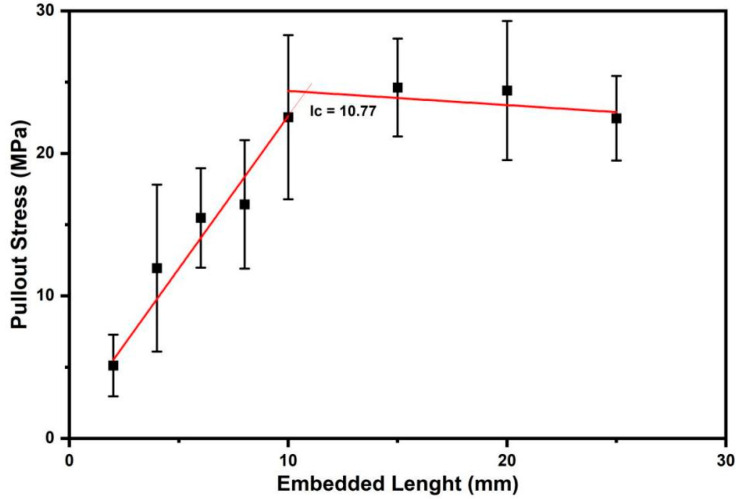
Comparison of pullout strength behavior with increasing embedded length of sedge fibers.

The pullout results allowed the construction of the graph in Figure 7. In this graph, two linear stages can be observed, which is a behavior that accords with the model proposed by Kelly and Tyson [33]. In the first stage, the tensile strength increases linearly with the embedded length of the fiber in the epoxy resin matrix. When this tensile strength reaches the limit stress of the fiber, its rupture occurs. The embedded length at the end of this stage is known as the critical length (*Ic*). For lengths less than *Ic*, complete interfacial detachment occurs, while for higher lengths, fiber failure occurs without disrupture at the fiber and matrix interface. The value of the lc determines whether the fiber is long enough to act as reinforcement or is just an embedded load. In the latter scenario, there is no load transfer from the matrix to the fiber [53]. 

From the linear stages shown in the plot in Figure 7 for tensile stress against embedded length, Equations (6) and (7) were adjusted.

(6) $$\sigma_{f}=1.22713+2.14111.L$$

(7) $$\sigma_{f}=25.38711-0.09932.L$$

The intersection of Equations (6) and (7) defines the critical length (*Ic*) of the sedge fibers being reached when the value is 10.77 mm. From this *Ic* value, the value of the fiber/matrix interfacial bond strength (*τc*) can be obtained. The average fiber diameter value of 0.675 mm and the average tensile strength value of 32.35 MPa were used.

(8) $$\;\tau c=\frac{0.675\mathrm{mm}\;\times 32.35\;\mathrm{MPa}}{10.77\;\mathrm{mm}\;\times 2}\;\;\;\to \;\tau c=1.014\;\mathrm{MPa}$$

Considering other values for different NLFs present in the literature, one can assume that an interface of sedge fiber and an epoxy matrix is comparatively weak: other interfaces, such as curaua/polyester with a value of 3.4, coir/polyester with a value of 3.8, ramie/polyester with 6.2, and sisal/polyester with 7.3, are comparatively stronger [53]. Moreover, Table 6 depicts the comparison of sedge fibers interfacial strength with other NLFs reported in literature [54,55] that were embedded in epoxy resins. Another factor that can influence an interface in composites and fibers is their interaction, including the volume of interaction between the fiber and the matrix [56]. In general, the interfacial interaction of a filler with a polymer matrix plays an effective role in the properties of composites [57]. However, under impact conditions, a low interfacial bond strength promotes a fiber/matrix generating longitudinal crack propagation, directly associated with higher impact energy and higher impact strength [58]. 

It can be seen in Table 6 that the coir and PALF fibers displayed a comparatively stronger interfacial interaction when compared to sedge fiber. However, under impact conditions, a low interfacial bonding strength provides delamination between the fiber/matrix interface by the longitudinal propagation of cracks, directly associated with greater impact energy and greater impact resistance, as reported by Neuba et al. [22].

### 3.6. Interfacial Observation

**Figure 8:**
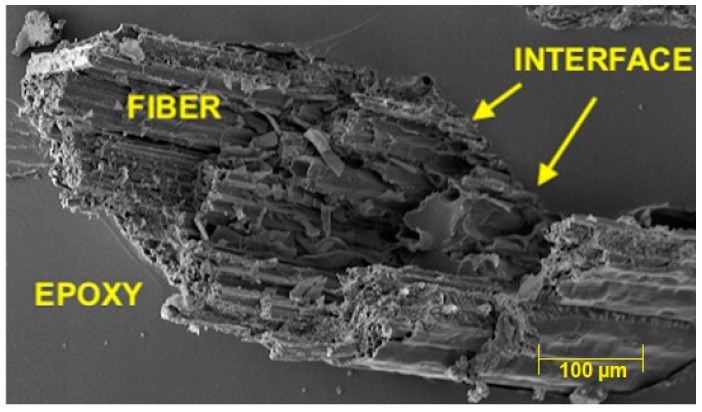
Micrograph of the fracture surface of a sedge fiber embedded in epoxy matrix.

Figure 8 shows an example of a sedge fiber embedded into an epoxy matrix. Despite a comparatively weaker *τc*, there was good fiber/epoxy interfacial bonding with only few open voids, which prevented fiber pullout from the matrix.

## 4. Conclusions

A crystallinity index (*Ic*) of 62.47% was obtained by X-ray diffraction (XRD) in samples of sedge fiber and is within the range of values reported for several natural lignocellulosic fibers (NLFs). Microfibril angle (MFA) of 7.36° was found by XRD for the sedge fiber sample. The relatively low MFA of sedge fiber is coherent with a superior tensile strength of 168.48 MPa. 

A Weibull statistical analysis of the ultimate tensile strength values revealed an inverse correlation between the sedge fiber mean diameter and the tensile strength. The sedge fibers exhibited a similar tensile strength when compared to NLFs from the same botanical gender. For the tensile test, ANOVA and Tukey statistical analyses proved, with a 95% confidence level, that the tensile stress increased as the diameter of the fibers decreased. 

Scanning electron microscopy (SEM) analysis provided evidence that thicker sedge fibers could have lower mechanical properties and undergo a premature rupture since they present more hollow spaces inside of the microfibrils that act as a mechanism of rupture. Statistically, in thicker fibers, there is a larger distribution of fibrils, allowing the weakest fibrils to break shortly when compared to thinner fibers that have fewer fibrils. 

The sedge presented in the pullout test has a critical length of 10.77 mm, which indicates weaker interfacial adhesion between this fiber and epoxy matrix, Also the value of interfacial strength was 1.014 MPa and was comparatively lower than other NLFs investigated in the literature. However, SEM revealed good fiber/epoxy bonding preventing the sedge fiber to pullout.

## Figures and Tables

**Table 1:** Weibull parameters for tensile strength of sedge fibers associated with different mean diameters.

Mean Diameter (mm)	Tensile Strength (MPa)	Statistical Deviation (MPa)	Weibull Modulus (*β*)	Characteristic Stress. *θ* (MPa)	Precision Adjustment (R²)
0.23	163.48	84.88	2.07	189.40	0.97
0.36	81.73	14.28	6.68	87.26	0.88
0.48	66.02	47.83	1.47	76.67	0.92
0.61	32.35	28.27	0.46	46.94	0.76
0.74	61.83	33.12	1.99	71.33	0.97
0.86	40.74	20.66	2.13	47.35	0.75

**Table 2:** Comparison of sedge fibers tensile strength with different NLFs and synthetic fibers.

Fiber	Tensile Strength (MPa)	References
Sedge	75	PW *
*Saccharum Bengalense*	33	[44]
Jute	393–773	[45]
Sisal	511–635	[45]
Kenaf	930	[45]
Piassava	133	[46]
Coir	106–270	[46]
Flax	1035	[47]
Curaua	543	[46]
Carbon	2500–5600	[48]
E-glass	2000–3500	[47]
Aramid	3000–3150	[47]

PW = Present work *.

**Table 3:** ANOVA results of average ultimate tensile strength obtained for seven-islands-sedge of for different mean diameters intervals.

Variation Causes	Sum of Squares	DF	Average Square	F (Calculated)	F_critical_ (Tabulated)
**Among the groups**	53,478.08	5	10,695.62	7.86	2.62
**Inside the groups**	32,641.36	24	1360.06		
**Total**	86,119.44	29			

**Table 4:** HSD test for the mean values of each diameter interval after application of the Tukey test.

Diameter Interval	1°	2°	3°	4°	5°	6°
1°	0	**78.67**	**88.89**	**124.72**	**110.72**	**119.65**
2°	**78.67**	0	10.32	46.05	32.10	40.97
3°	**88.89**	10.32	0	35.72	21.77	30.65
4°	**124.72**	46.05	35.72	0	13.95	5.07
5°	**110.72**	32.10	21.77	13.95	0	8.88
6°	**119.65**	40.97	30.65	5.07	8.88	0

**Table 5:** Maximum elongation and modulus of elasticity of the sedge of each diameter interval.

Diameter Intervals (mm)	Maximum Elongation (%)	Tensile Modulus of Elasticity (GPa)
0.23	4.08	4.36
0.36	3.56	2.96
0.48	4.22	1.92
0.61	2.79	1.79
0.74	4.07	1.77
0.86	4.49	0.96

**Table 6:** Comparison of sedge fibers interfacial strength with different NLFs embedded in epoxy resins.

Fiber	τc (MPa)	References
Sedge	1.014	PW *
Betelnut	0.880	[54]
coir	0.270	[55]
Fique	1.420	[55]
PALF	4.930	[55]

PW = Present work *.

## Data Availability

The data presented in this study are available on request from the corresponding author.

## Sample Availability

Samples of the compounds are available from the authors.
